# Characterizing the Impact of Primer-Template Mismatches on Recombinase Polymerase Amplification

**DOI:** 10.1016/j.jmoldx.2022.08.005

**Published:** 2022-09-16

**Authors:** Matthew Higgins, Oliver W. Stringer, Daniel Ward, Jennifer M. Andrews, Matthew S. Forrest, Susana Campino, Taane G. Clark

**Affiliations:** ∗ Faculty of Infectious and Tropical Diseases, London School of Hygiene and Tropical Medicine, London, United Kingdom; † Section of Paediatric Infectious Disease, Department of Infectious Disease, Imperial College London, London, United Kingdom; ‡ Wolfson College, University of Cambridge, Cambridge, United Kingdom; § Faculty of Epidemiology and Population Health, London School of Hygiene and Tropical Medicine, London, United Kingdom

## Abstract

Recombinase polymerase amplification (RPA) is an isothermal amplification assay that has been ubiquitously utilized in the detection of infectious agents. Like any nucleic acid amplification technology, primer-template complementarity is critical to RPA reaction success. Mismatches arising in the primer-template complex are known to impact reaction kinetics, invalidate downstream analysis, such as nucleic acid quantification, and result in false negatives if used in a diagnostic capacity. Although the impact of specific primer-template mismatches has been well characterized for techniques such as PCR, characterization remains limited for RPA. Through our study, we systematically characterize the impact of mismatches on the RPA reaction, when located in the 3′-anchor region of the primer-template complex. Our investigation identified that the nucleotides involved, as well as position of each mismatch, influence the size of the impact, with terminal cytosine-thymine and guanine-adenine mismatches being the most detrimental. The presence of some mismatch combinations, such as a penultimate cytosine-cytosine and a terminal cytosine-adenine mismatch pairing, led to complete RPA reaction inhibition. Through the successful characterization of 315 mismatch combinations, researchers can optimize their RPA assay accordingly and seek to implement RPA technology for rapid, in-field genotyping.

Recombinase polymerase amplification (RPA) is an isothermal nucleic acid amplification technique (NAAT) that has been ubiquitously implemented in the detection of human and plant pathogens.^1^^,^^2^ The RPA system relies on three T4 phage proteins, UvsX, UvsY, and Gp32. UvsX and UvsY facilitate priming of the DNA target through the assembly of a nucleoprotein filament, and Gp32 stabilizes the displaced single-stranded DNA during D-loop formation.^3^ Together, this equates to the denaturation and primer annealing steps of a typical PCR cycle. RPA's performance at 37°C to 42°C makes it ideal for use in low-resource field settings, as demonstrated during the 2015 Ebola outbreak.^4^ Unlike other common NAATs, such as PCR and loop-mediated isothermal amplification,^5^, ^6^, ^7^ under certain conditions, RPA can be performed in the absence of a heat block,^5^ highlighting its potential as the basis for future diagnostics.

RPA reaction success depends on robust primer design, like any NAAT.^8^ This process balances several factors, which include the following: ensuring primer specificity by maximizing Watson-Crick nucleotide base pairing,^9^ minimizing the potential of off-site binding to nontarget DNA, and minimizing primer-derived secondary structures, which can impede the reaction with varying degrees of severity.^10^ Technique-specific primer design software has been developed, such as PrimedRPA, Primer3, and PrimerExplorer, to assist this task and overcome limitations associated with manual primer design.^8^^,^^11^ However, the presence of unknown polymorphic sites in the primer binding region can compromise an NAAT reaction, causing nucleotide mismatches and reducing the stability of the primer-template complex.^12^^,^^13^ This reduction is particularly an issue for organisms with high genetic diversity, limited genomic characterization, or error-prone replication systems.^14^ Previous work has shown how mismatches can acutely disrupt NAAT amplification.^15^^,^^16^ Initial reports highlighted that RPA has a high tolerance to polymorphisms in the primer/probe binding sites,^17^ but subsequent work has identified that primer-template mismatches can impact RPA reaction efficiency and success, although they were unable to predict the impact based on the presence of a single mismatch.^18^ Single or multiple mismatches located toward the 3′ terminus of the primer result in the most severe disruption for PCR, significantly reducing amplification efficiency and, in certain cases, preventing amplification altogether.^15^ However, this phenomenon can be utilized to facilitate single-nucleotide polymorphism (SNP) genotyping, as demonstrated by amplification-refractory mutation system–PCR, kompetitive allele-specific PCR, and other techniques.^19^^,^^20^ The genotyping of informative SNPs can personalize treatment choices and inform related fields, such as pharmacogenomics, where these biomarkers can be integrated into treatment decision pathways.^21^ For example, several SNPs have been identified in metabolic genes that confer increased sensitivity or tolerance to the widely used warfarin anticoagulant drug,^22^ and whose detection can inform the correct dosing in individuals with a high risk of thromboembolism, lowering the risk of adverse drug events due to underlying xenobiotic metabolism heterogeneity.^23^, ^24^, ^25^

This study aims to build on previous work and systematically characterize the impact of mismatches on the RPA reaction across four human genetic loci with clinical relevance to warfarin treatment. Two loci are situated within the *cyp2c9* gene, linked to warfarin clearance, whereas the other two loci are found within genes associated with altered warfarin dosage levels (*vkorc1* and *hbb*). The study centers on mismatches located in the primer anchor region, defined as the pre-ante-penultimate to 3′-terminal position, and attempts to build a mismatch classification system that can predict the impact of a given mismatch on RPA reaction success and kinetics. For this investigation, formamidopyrimidine DNA glycosylase (fpg) probes were utilized as, unlike the commonly used exo probes, they cannot act as extendable primers after cleavage and as such do not influence reaction kinetics. In addition, the fpg enzyme utilized for fpg probe cleavage has no 3′-5′ exonuclease activity, which could reduce the length of the primers removing mismatch loci under investigation. Understanding the impact on reaction kinetics is vital in determining whether the presence of a given mismatch will compromise techniques, which rely on the kinetic profile, such as RPA-based nucleic acid quantification.^26^ Furthermore, this characterization could aid in the adaptation of RPA for in-field rapid SNP genotyping.

## Materials and Methods

### Chemicals and Oligonucleotide Design

All RPA reactions were performed using the TwistAmp fpg kit (TwistDx Ltd., Cambridge, UK). Oligonucleotide primers were sourced from Eurogentec (Seraing, Belgium) and TwistAmp fpg probes from LGC Biosearch Technologies (Petaluma, CA). Four nonsynonymous SNPs linked to warfarin metabolic changes were identified (Table 1). The double-stranded DNA templates housing each locus were procured from Twist Bioscience (San Francisco, CA) and subsequently diluted to the desired copy number in Tris-EDTA with 1 ng/μL poly(2′-deoxyinosinic-2′-deoxycytidylic acid) sodium salt (Table 2). Seven assays were designed, targeting the four selected loci (Table 3). For each assay, the SNP was located in a primer binding region, with 52 dynamic primer variants generated through the exchange of one or two nucleotides from the pre-ante-penultimate to the 3′ terminal position (Table 3). As such, the impact of a single and/or combined mismatches on amplification could be studied, while accounting for their relative position.

**Table 1:** Loci of Interest

Gene	Gene orientation	Wild type (+/–)	Mutation (+/–)	SNPedia identifier	Description
*cyp2c9*	+	(A/T)	(C/G)	*rs1057910*	*cyp2c9* Encodes a member of the cytochrome P450 superfamily of enzymes, which is a key component in the xenobiotic metabolism of warfarin. This mutation has been shown to decrease enzyme activity, reducing rates of warfarin clearance and, as such, increasing sensitivity.^25^
*cyp2c9*	+	(G/C)	(A/T)	*rs4244285*	This mutation produces a nonfunctional truncated enzyme. Subsequently, the rate of warfarin clearance is reduced, and individuals have increased sensitivity.^25^
*vkorc1*	+	(C/G)	(G/C)	*rs8050894*	Warfarin inhibits the enzyme activity of vitamin K epoxide reductase complex C1 encoded by the *vkorc1* gene. The mutation highlighted confers resistance and, as such, a higher dose of warfarin is required for effective treatment.^27^
*hbb*	–	(T/A)	(A/T)	*rs334*	Carriers of the homozygous *TT* genotype develop sickle cell disease, increasing the underlying risk of blood clots. Low-dose warfarin treatment has been shown to reduce the risk of adverse effects linked to clotting.^28^

**Table 2:** dsDNA Templates

SNP	Sequence
>*rs105791*	5′-TTTAAGTTTGCATATACTTCCAGCACTATAATTTAAATTTATAATGATGTTTGGATACCTTCATGATTCATATACCCCTGAATTGCTACAACAAATGTGCCATTTTTCTCCTTTTCCATCAGTTTTTACTTGTGTCTTATCAGCTAAAGTCCAGGAAGAGATTGAACGTGTGATTGGCAGAAACCGGAGCCCCTGCATGCAAGACAGGAGCCACATGCCCTACACAGATGCTGTGGTGCACGAGGTCCAGAGATACNTTGACCTTCTCCCCACCAGCCTGCCCCATGCAGTGACCTGTGACATTAAATTCAGAAACTATCTCATTCCCAAGGTAAGTTTGTTTCTCCTACACTGCAACTCCATGTTTTCGAAGTCCCCAAATTCATAGTATCATTTTTAAACCTCTACCATCACCGGGTGAGAGAAGTGCATAACTCATATGTATGGCAGTTTAACTGGACTTTCTCTTGTTTCCAGTTTGGGGCTATAAAGGTTTGTAACAGGTCCTAGTGTCTGGCAGTGTGTGTTCTCCAGATTTATTATCTTTCTTCAAGATTGGTTTGGCTACTCTTAGGTGCTTATATTTCCAAATAATT-3′
>*rs334*	5′-GCACTTTCTTGCCATGAGCCTTCACCTTAGGGTTGCCCATAACAGCATCAGGAGTGGACAGATCCCCAAAGGACTCAAAGAACCTCTGGGTCCAAGGGTAGACCACCAGCAGCCTAAGGGTGGGAAAATAGACCAATAGGCAGAGAGAGTCAGTGCCTATCAGAAACCCAAGAGTCTTCTCTGTCTCCACATGCCCAGTTTCTATTGGTCTCCTTAAACCTGTCTTGTAACCTTGATACCAACCTGCCCAGGGCCTCACCACCAACTTCATCCACGTTCACCTTGCCCCACAGGGCAGTAACGGCAGACTTCTCC**N**CAGGAGTCAGATGCACCATGGTGTCTGTTTGAGGTTGCTAGTGAACACAGTTGTGTCAGAAGCAAATGTAAGCAATAGATGGCTCTGCCCTGACTTTTATGCCCAGCCCTGGCTCCTGCCCTCCCTGCTCCTGGGAGTAGATTGGCCAACCCTAGGGTGTGGCTCCACAGGGTGAGGTCTAAGTGATGACAGCCGTACCTGTCCTTGGCTCTTCTGGCACTGGCTTAGGAGTTGGACTTCAAACCCTCAGCCCTCCCTCTAAGATATATCTCTTGGCCCCATACCATCAGTACAAATTGCTACTAAAAACATCCTCCTTTGCAAGTGTATTTACGTAATATTTGG-3′
>*rs424428*	5′-ACCATCTTATATTTCAAGATTGTAGAGAAGAATTGTTGTAAAAAGTAAGAGAATTAATATAAAGATGCTTTTATACTATCAAAAGCAGGTATAAGTCTAGGAAATGATTATCATCTTTGATTCTCTTGTCAGAATTTTCTTTCTCAAATCTTGTATAATCAGAGAATTACTACACATGTACAATAAAAATTTCCCCATCAAGATATACAATATATTTTATTTATATTTATAGTTTTAAATTACAACCAGAGCTTGGCATATTGTATCTATACCTTTATTAAATGCTTTTAATTTAATAAATTATTGTTTTCTCTTAGATATGCAATAATTTTCCCACTATCATTGATTATTTCCC**N**GGAACCCATAACAAATTACTTAAAAACCTTGCTTTTATGGAAAGTGATATTTTGGAGAAAGTAAAAGAACACCAAGAATCGATGGACATCAACAACCCTCGGGACTTTATTGATTGCTTCCTGATCAAAATGGAGAAGGTAAAATGTTAACAAAAGCTTAGTTATGTGACTGCTTGCGTATTTGTGATTCATTGACTAGTTTTGTGTTTACTACGGATGTTTAACAGGTCAAGGAGTAATGCTTGAGAAGCATATTTAAGTTTTTATTGTATGCATGAATATCCAGTAAGCATCATAGAAAATGTAAAATTAAAT-3′
>*rs8050894*	5′-ACATGGCGAGACACCATCTCTACCAAAAAAAAACAAAAACAAAAATTAGCTGGGCATAGTGGTGCACGCCTGTGATTCCAGCTGCTTGGGAGGCTAAGGTGGGAGGATCCCTTGGGCAGGGAGGCAGAGGTTGCCATGAACTGAGATCACGCCAGTGCACACTAAGGGCATCCTAGACCTCACTTTGGGCAACAGAGCCAGACCCTGTCTCAAAACAACAACAAACAAAAAACCTGGGGACCTAGGATGTCTTTAAGGGCCCTTCAGCCTCTAACAGTACTTAAACCAATTAAAAGACTCCTGTTAGTTACCTCCCCACATCCCCACCCGCAGGACGCTC**N**GTGATGAGCAGCTAGCTGGCTGTCAGCTGTGTGGATCACCAAGATTGCATGGAGTGGGGCTGAGCTGACCAAGGGGGATGAGGGGCGGGGCGGGGCGGGCAGGGAGGGGGCGGAGCCACTCACCTAACAATAGCTGTAGTGTGTAGAAGATGCAACCGAATATGCTGTTGGATTGATTGAGGATGCTGTCCTGTCCCAGCACATGCTCCACCAGCCCGAAACCCCTGCCCCACCTGGCAGAGGGGTGGGGTGGGGTGGAACCAGGTTAGGACTGTCAACCCAGTGCCTTGGACCCTGCCCGAGAAAG-3′

These sequences were procured from Twist Bioscience. The N value (in bold) indicates the single-nucleotide polymorphism (SNP) site that was modified to generate four template variants per loci.

dsDNA, double-stranded DNA.

**Table 3:** RPA Primers and Probe Groups Used in This Investigation

Group	Target	Sense	Role	Sequence
1	RS4244285	–	FP	5′-AAATTACAACCAGAGCTTGGCATATTGTATCTATA-3′
1	RS4244285	–	RP	5′-GCAAGGTTTTTAAGTAATTTGTTATGGGT**TCCN**-3′
1	RS4244285	–	Probe	5′-TCTTAGATATGCAATAATTTTCCCACT(dR[FAM])TCA(dT[BHQ-1])TGATTATTTCC-3′
2	RS1057910	–	FP	5′-ATCAGCTAAAGTCCAGGAAGAGATTGAACGTGTGA-3′
2	RS1057910	–	RP	5′-GCATGGGGCAGGCTGGTGGGGAGAAGGT**CAAN**-3′
2	RS1057910	–	Probe	5′-TGGCAGAAACCGGAGCCCCTGCATGCAA(dR[FAM])ACAG(dT[BHQ-1])AGCCACATG-3′
3	RS8050894	–	FP	5′-CTTCAGCCTCTAACAGTACTTAAACCAATTA-3′
3	RS8050894	–	RP	5′-CACACAGCTGACAGCCAGCTAGCTGCTCAT**CACN**-3′
3	RS8050894	–	Probe	5′-[FAM]AA[dR-BHQ-1]ACTCCTGTTAGTTACCTCCCCACATCC-3′
4	RS334	–	FP	5′-CATCTATTGCTTACATTTGCTTCTGACACAAC-3′
4	RS334	–	RP	5′-CCCACAGGGCAGTAACGGCAGACTTC**TCCN**-3′
4	RS334	–	Probe	5′-CAGGAGTCAGATGCACCATGGTGTCT(dR[FAM])TT(dT[BHQ-1])GAGGTTGCTAGT-3′
5	RS4244285	+	FP	5′-ATAATTTTCCCACTATCATTGATTATTT**CCCN**-3′
5	RS4244285	+	RP	5′-CTTTTGTTAACATTTTACCTTCTCCATTTTGAT-3′
5	RS4244285	+	Probe	5′-CACTTTCCATAAAAGCAAGGTTTTTAA(dR[FAM])TAA(dT[BHQ-1])TTGTTATGGGT-3′
6	RS1057910	+	FP	5′-AGATGCTGTGGTGCACGAGGTCCAGAGA**TACN**-3′
6	RS1057910	+	RP	5′-CAGTGTAGGAGAAACAAACTTACCTTGGGAATGAGA-3′
6	RS1057910	+	Probe	5′-TTAATGTCACAGGTCACTGCATGGGGCAGGCT(dR[FAM])G(dT[BHQ-1])GGGGAGAAGGT-3′
7	RS334	+	FP	5′-CAACCTCAAACAGACACCATGGTGCATCTGACTC**CTGN**-3′
7	RS334	+	RP	5′-GCCCAGTTTCTATTGGTCTCCTTAAACCTGTCTTG-3′
7	RS334	+	Probe	5′-CTGCCGTTACTGCCCTGTGGGGCAA(dR[FAM])G(dT[BHQ-1])GAACGTGGATGAA-3′

Bold indicates dynamic primer variants; N-terminal nucleotide covers loci of interest.

FP, forward primer; RP, reverse primer; RPA, recombinase polymerase amplification.

### RPA Amplification

All reactions followed the recommended TwistAmp fpg protocol. A total of 600 nmol/L of forward and reverse primers, 120 nmol/L of probe, DNA template, 1× rehydration buffer, and DNAse-free water to a total volume of 47.5 μL were added to each lyophilized TwistAmp fpg pellet. A clean 2-mm bearing ball was then added to each tube to allow magnetic mixing to occur. Reactions were simultaneously initiated through the addition of 2.5 μL of 280 mmol/L magnesium acetate to the lids of the reaction tubes (strip of eight), capping the tubes carefully and spinning the magnesium acetate into the rehydrated material (total reaction volume 50 μL). Reactions were vortexed briefly and spun down once again before being placed into T8-ISO fluorescence readers manufactured by Axxin (Melbourne, VIC, Australia). Reactions were run at 39°C for 20 minutes, with readings taken every 10 to 15 seconds with an Opto PWM Duty FAM setting of 17 or 20.

### Mismatch Characterization

For each assay, the reliable limit of detection (rLOD) was established (1000 to 5000 copies) in the absence of mismatches. The assessment of all following dynamic primers proceeded with two technical replicates against each relevant template variant. Primers resulting in a lone 3′-terminal mismatch were assessed against 1×, 10×, and 1000× the rLOD, whereas primers resulting in a lone internal mismatch were assessed at 1× the rLOD. Finally, primers that introduced multiple mismatches were assessed at 100× and 500× the rLOD. In each experiment, a primer with full complementarity to the target site was included to act as an internal standard and assessed at 1× the rLOD. Multiples of the rLOD were used in mismatch reactions as previous work has shown the introduction of mismatches reduced reaction sensitivity.^15^, ^16^ Mismatches were classified categorically according to the nucleotides present in the anchor region, disregarding complementary base-pairing positions. For example, the following primer (5′--CCCT′)–template (3′--GAGT) complex anchor region would be categorized as (?C?T-?A?T). In total, our data set covered 315 unique primer-template mismatch combinations.

### Reaction Kinetic Profiling and Thermodynamic Calculations

**Figure 1:**
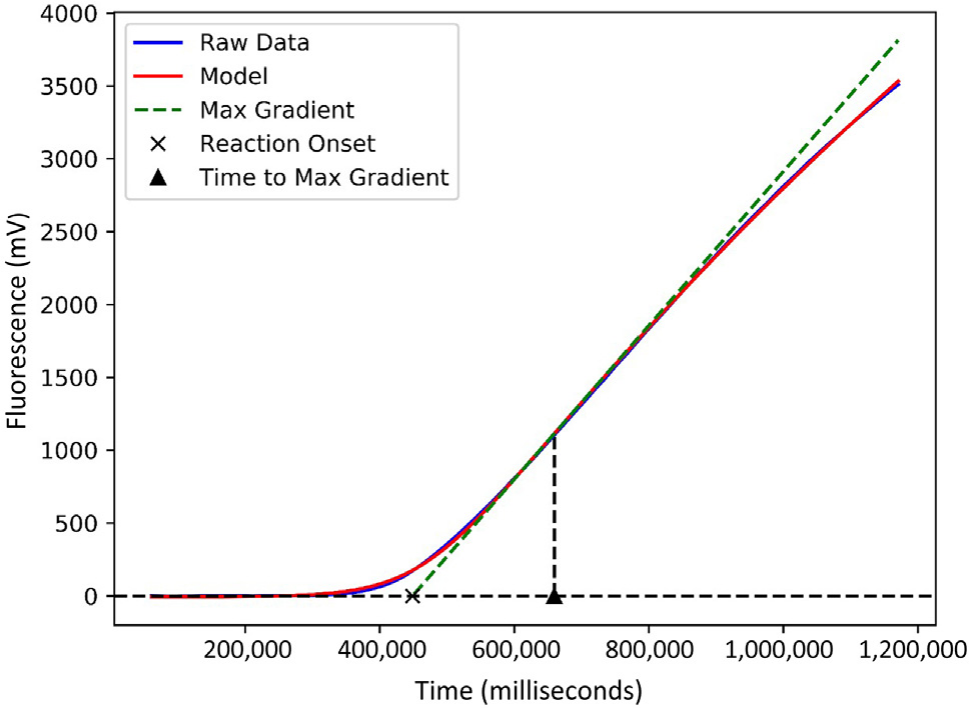
Modeling of the fluorescent curve after baseline normalization via a generalized logistic function and subsequent derivation of reaction kinetic metrics. The fluorescence profile corresponds to primer-probe group 6 ???C-???T terminal mismatch. Max, maximum.

The fluorescence profile of each reaction was extracted from the T8-ISO output, and a custom python script was used to normalize all reactions against their respective baseline (*https://github.com/MatthewHiggins2017/RPALogisticModelling*, last accessed August 25, 2022). Reaction success was established through a standard minimum fluorescence threshold criterion. Each successful reaction was modeled using a generalized logistic function (Richards' curve), via the Scipy python package (*https://scipy.org*, last accessed August 25, 2022). For each reaction, the time to positivity (TP), maximum gradient (MG), and time to maximum gradient (TMG) were derived (Figure 1). The 3′ Gibbs free energy was determined for each mismatch combination, according to the nearest-neighbor thermodynamic model using the full anchor region sequence. This model used prederived values, which are validated under crowding conditions present in the RPA reaction.^29^^,^^30^ Calculations were performed using a custom python script.

### Statistical Analysis

To explore the impact of mismatch(es) on the odds of RPA reaction success, Firth logistic regression was implemented using the logistf package in R statistical software version 4.1.3 (*https://www.r-project.org*). This method was selected on the basis of its ability to handle complete separation events, such as a given mismatch combination inhibiting the RPA reaction across all experiments. In addition, Firth logistic regression can account for representational imbalances in the data set, which exist for primers that introduced two mismatches, due to limitations imposed by the nucleotides adjacent to the SNP of interest, which remained unadjusted in our double-stranded DNA templates. Area under the receiver-operating characteristic curve (AUC) analysis was implemented to assess model performance.

The impact of mismatches on successful reaction RPA kinetics (TP, MG, and TMG) was investigated using a robust linear mixed model, using the robustlmm R package.^31^ The mixed model allowed us to account for the hierarchical data structure introduced as experiments were discretely clustered according to the primer-probe groups. Inclusion of a random effect variable for experiment accounted for human introduced variation, which could arise, including subtle time delays between the addition of magnesium acetate to start the reaction to placing the eight-tube strip in the fluorescence reader. Inclusion of a random effect variable for the target accounted for intrinsic performance differences between the seven assays, which could be linked to amplicon length, secondary structure formation, and a range of other factors beyond the scope of this investigation. The fixed variable, Opto PWM Duty FAM, was included in the model to account for calibration differences between the T8-ISO fluorescence readers utilized. A robust approach was chosen because of the heavy tailed residual distribution, ensuring all assumptions associated with statistical inference were met. To assess mixed model performance, conditional *R*^2^ values were derived according to Nakagawa and Schielzeth.^32^ In all models, the baseline level of the mismatch categorical variable (????-????) represented complete complementary binding of the anchor region, allowing us to compare the impact of primer-template mismatches on the RPA reaction against primer binding with complete complementarity.

## Results

### First Models for RPA Reaction Success and Kinetics

Across 501 experiments covering 315 unique mismatch combinations, a total of 3543 reactions of 4008 were classified as successful (88.4%). The model established to investigate mismatch impact on reaction success achieved an AUC of 0.88 (Supplemental Table S1 provides estimated coefficients). More than 150 mismatch combinations (compared with ????-????) were identified to have a significant impact on reaction success (159 with *P* < 0.05), representing 50.4% of all mismatches investigated. The double-stranded DNA template copy number was found not to have a significant impact on the probability of reaction success (*P* = 0.549). The models derived for the TP, MG, and TMG achieved conditional (and adjusted for model size) *R*^2^ values of 0.867 (0.854), 0.862 (0.850), and 0.800 (0.781), respectively. The template copy number had a significant impact across all three reaction kinetic metrics (TP, MG, and TMG), where a unit increase in the copy number resulted in a decrease in TP and TMG, while increasing the MG (*P* < 0.001) (Supplemental Table S1). Overall, 252, 188, and 250 mismatches (compared with ????-????) had a significant (*P* < 0.05) impact on TP, MG, and TMG, respectively. In total, 106 mismatches significantly impacted all the reaction kinetics metrics, as well as the probability of reaction success.

### The Impact of Primer-Template Mismatch Constituents on RPA Reaction Kinetics

**Figure 2:**
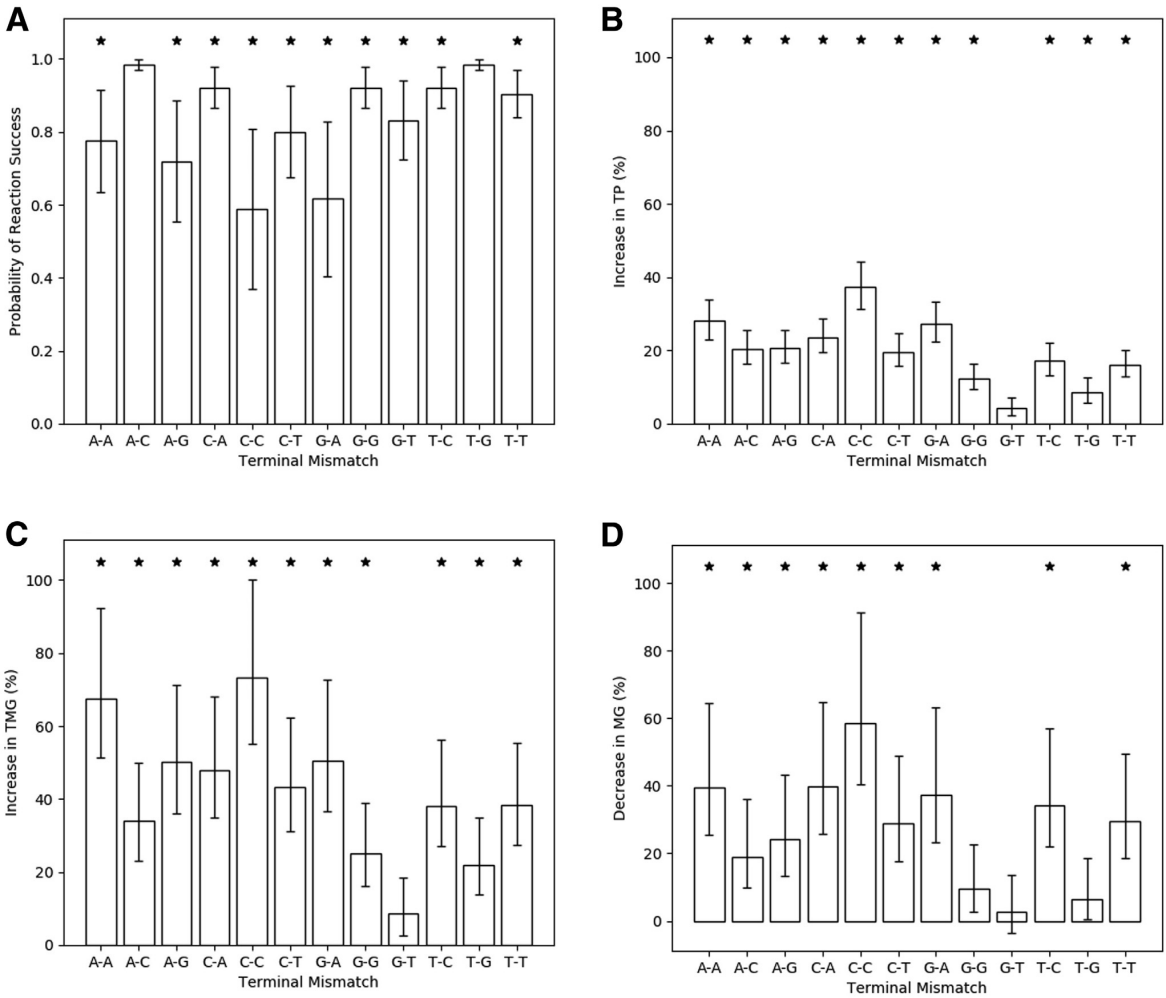
Impact of terminal mismatches on recombinase polymerase amplification reaction success (**A**); increase in time to positivity (TP; **B**); increase in time to maximum gradient (TMG; **C**); and decrease in maximum gradient (MG; **D**) when compared with primer-template complexes with complete complementarity. ∗*P* < 0.05.

**Figure 3:**
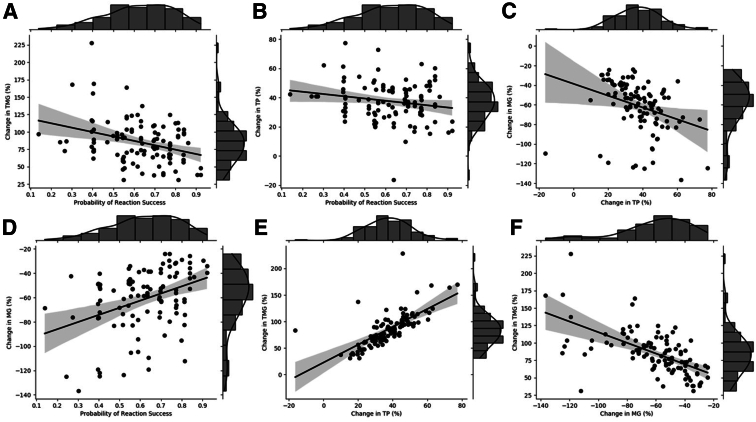
Spearman correlation plots for recombinase polymerase amplification reaction metrics of interest. For each comparison, linear regression was used to establish a line of best fit (solid black) and the associated 95% CI (shaded gray) **A:** Change in time to maximum gradient (TMG) versus probability of reaction success. **B:** Change in time to positivity (TP) versus probability of reaction success. **C:** Change in maximum gradient (MG) versus change in TP. **D:** Change in MG versus probability of reaction success. **E:** Change in TMG versus change in TP. **F:** Change in TMG versus change in MG.

To investigate whether the constituents of a given primer-template mismatch alter the impact on RPA kinetic profile, the impact of single terminal mismatches was studied. This impact was assessed when expressing model coefficients relative to primer-template complexes with complete complementary binding (Figure 2). Of the 12 possible terminal mismatches, 8 resulted in a significant difference (*P* < 0.05) across all reaction metrics, and a quarter of all possible terminal mismatches resulted in the probability of reaction success decreasing below 0.8. The impact of a given mismatch deviates depending on the nucleotide constituents. The cytosine-cytosine terminal mismatches (primer-template) appear to be most detrimental to the probability of reaction success (0.59), closely followed by the guanine-adenine terminal mismatch (0.62). This pattern continues for the increase in TP for cytosine-cytosine (37.3%) and guanine-adenine (27.4%) terminal mismatches. The cytosine-cytosine terminal mismatch also results in the largest decrease in MG (58.4%) and increase in TMG (73.2%). Only two terminal mismatches do not significantly impact the probability of reaction success, thymine-guanine (0.98) and adenine-cytosine (0.98). The only terminal mismatch to not significantly affect any of the reaction kinetics is guanine-thymine (primer-template), but this was found to have a significant impact on the probability of reaction success (0.83). The pairwise Spearman correlation between each reaction metric was determined, considering only those mismatches that resulted in a significant impact across all metrics (Figure 3 and Table 4). A significant pairwise correlation (*P* < 0.05) was found between all metrics apart from the probability of reaction success versus a change in TP. The strongest correlation (ρ: 0.823) was observed between the change in TP and TMG metrics, which is to be expected.

**Table 4:** Spearman Correlation Values Obtained between Different RPA Reaction Metrics

Metric 1	Metric 2	ρ	*P* value
Probability of reaction success	Change in TP (%)	−0.138	1.60 × 10^−1^
Probability of reaction success	Change in MG (%)	0.357	1.72 × 10^−4^
Change in TP (%)	Change in MG (%)	−0.489	1.04 × 10^−7^
Change in TP (%)	Change in TMG (%)	0.823	2.61 × 10^−27^
Probability of reaction success	Change in TMG (%)	−0.260	7.23 × 10^−3^
Change in MG (%)	Change in TMG (%)	−0.649	5.41 × 10^−14^

MG, maximum gradient; RPA, recombinase polymerase amplification; TMG, time to maximum gradient; TP, time to positivity.

### The Impact of Primer-Template Mismatch Location on RPA Reaction Success and Kinetics

**Figure 4:**
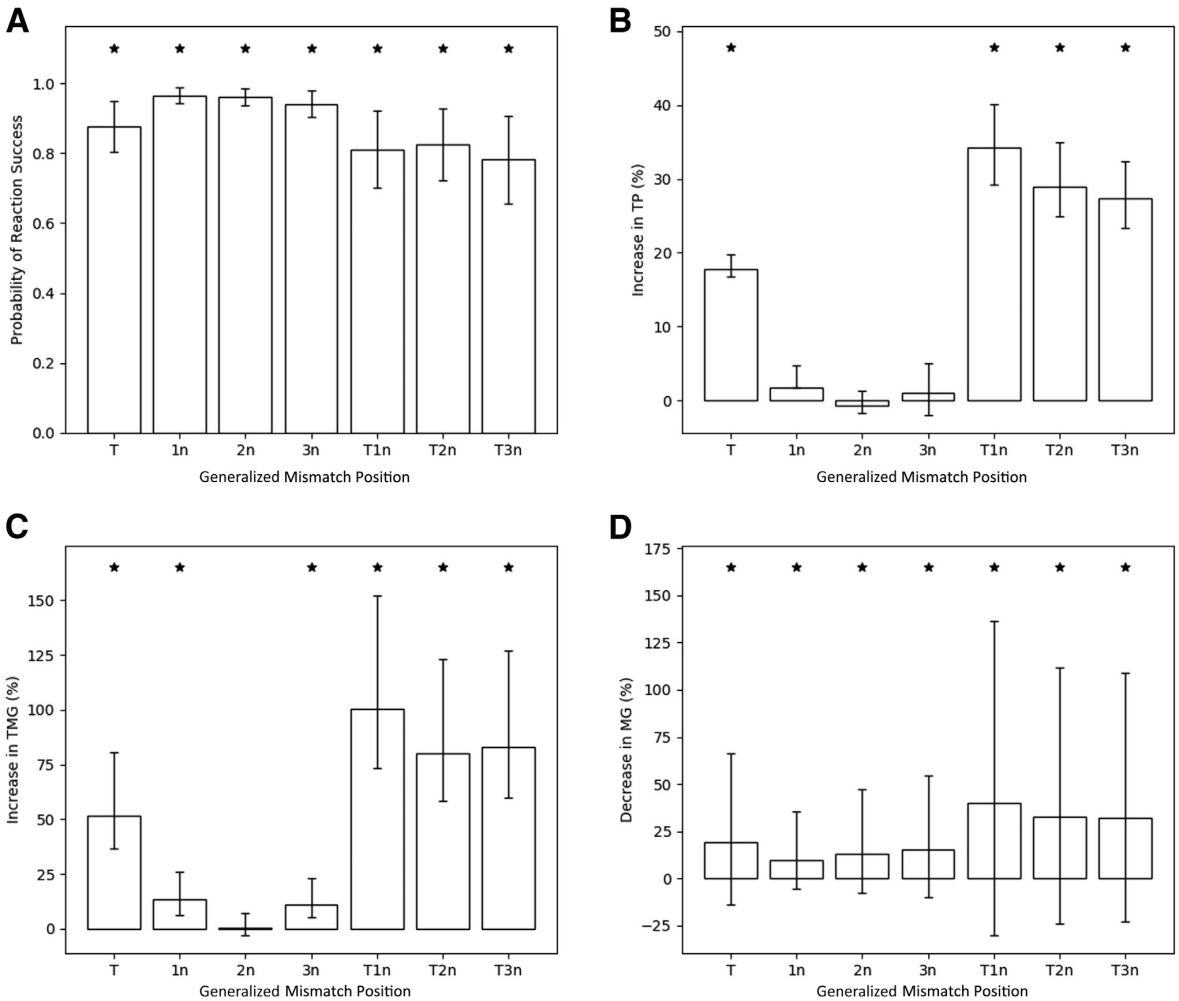
Positional impact of mismatches on probability of reaction success (**A**); increase in time to positivity (TP; **B**); increase in time to maximum gradient (TMG; **C**); and decrease in maximum gradient (MG; **D**) when compared with primer-template complexes with complete complementarity. ∗*P* < 0.05.

Next, the detrimental effect of a primer-template mismatch according to the relative positioning of mismatches in the anchor region was studied. Primer-template complexes were categorized into eight groups (T, 1n, 2n, 3n, T1n, T2n, and T3n; compared with P – ????-????) (Table 5). With this new classification system, models for each metric were fitted (Supplemental Table S2 provides estimated coefficients). The updated model for the probability of reaction success achieved an AUC of 0.72, whereas the updated mixed models for TP, MG, and TMG obtained conditional (and adjusted) *R*^2^ values of 0.792 (0.791), 0.826 (0.826), and 0.656 (0.655), respectively. The predictive performance of the updated models is inferior to those including unique mismatch combinations, but they contain fewer parameters. From a generalized standpoint, any mismatches positioned in the anchor region may have a significant impact on the probability of reaction success (Figure 4). When considering the positional impact of lone primer-template mismatches (T, 1n, 2n, and 3n), those located at the terminal position are the most detrimental, resulting in a 17.7% increase in TP, a 19.4% decrease in MG, and a 51.7% increase in TMG, with a probability of reaction success of 0.877 (Figure 4). Lone mismatches located outside of the terminal position do not have a significant impact on TP, whereas those located in the 1n and 3n positions do significantly (*P* < 0.05) impact MG (9.7% and 15.3%) and TMG (13.2% and 11.1%), respectively. The presence of two mismatches is more detrimental across all RPA reaction metrics than the presence of a lone mismatch, regardless of position. For TP and MG, the size of the impact decreases as the distance between the secondary mismatch and terminal mismatch grows. The impact on TP decreases from 34.17% to 27.35% and on MG from 40.13% to 32.10% for T1n and T3n, respectively. However, this is not the case for reaction success and TMG, where T3n and T1n appear to be the most detrimental, respectively.

**Table 5:** Reclassification of Primer-Template Complex to Investigate Positional Impact on RPA Reaction Success and Kinetics

New classification	Original classification	New classification sample size
P	????-????	1002
T	???A-???G	510
1n	??A?-??G?	186
2n	?A??-?G??	192
3n	A???-G???	192
T1n	??AA-??GG	624
T2n	?A?A-?G?G	630
T3n	A??A-G??G	672

RPA, recombinase polymerase amplification.

### The Impact of Anchor Region Stability on RPA Reaction Success and Kinetics

To determine whether the anchor region stability could be used to predict changes in RPA reaction success and kinetics, the Gibbs free energy for each primer-template mismatch was estimated. The thermodynamic potential reflects the stability of the primer-template complex anchor region and is currently a selection feature in popular primer design software (eg, PrimerExplorer and Primer3). Primer-template complexes with mismatches in the terminal and penultimate positions were excluded because of limitations of values available used to generate the nearest-neighbor thermodynamic model. The Gibbs free energy values were used to replace the categorical mismatch values in revised models for each metric (Supplemental Table S3 provides estimated coefficients). The AUC obtained for the probability of reaction success was 0.734, whereas the conditional (and adjusted) *R*^2^ values for TP, MG, and TMG were 0.658 (0.658), 0.857 (0.857), and 0.550 (0.550), respectively. Across all RPA reaction metrics, the Gibbs free energy variable had a significant impact (*P* < 0.05), where a unit increase in energy resulted in an increase in TMG and TP and a decrease in MG (Supplemental Table S3). These directions of effect were expected as an increase in the Gibbs free energy represents a decrease in anchor region stability.

To enable an accurate comparison to prior mismatch classification models, the positional-based classification models were refitted, excluding the T1n data (Supplemental Table S4). For the updated positional models, the probability of reaction success AUC was 0.748, whereas the *R*^2^ values for TP, MG, and TMG were 0.711 (0.710), 0.874 (0.874), and 0.600 (0.599), respectively. By comparing measures of model fit, utilizing adjusted-conditional *R*^2^ metric, the performance of using Gibbs free energy to the positional-based mismatch classification is similar. However, across all metrics, positional-based mismatch classification narrowly outperforms models that include the Gibbs free energy. In addition, using positional-based classification allows combined mismatches in the penultimate and terminal position of the primer-template mismatch complex to be accounted for.

## Discussion

Our investigation has shown the detrimental impact of primer-template mismatches on RPA amplification when located toward the 3′ primer terminus. To the best of our knowledge, this is the first study to systematically explore RPA kinetics via modeling the reaction fluorescence profile using a generalized logistic function. As expected, when classifying mismatches according to position, the presence of multiple mismatches resulted in a greater impact on the RPA reaction, compared with the presence of a lone mismatch. Multiple mismatches, positioned adjacently in the 3′ primer terminal and penultimate position, were found to be the most detrimental across all reaction kinetic metrics. Our analysis reveals that the impact of a given mismatch combination is not only dependent on the relative position in the anchor region but also the nucleotides involved. Most mismatch combinations significantly impacted at least one reaction measure, with just over a third of mismatches (106/315) significantly impacting all metrics considered. Specifically, a terminal cytosine-cytosine mismatch was the most detrimental to the RPA reaction efficiency, followed by a guanine-adenine. However, adenine-cytosine and thymine-guanine mismatches were highly tolerated, rarely resulting in amplification failure, mirroring the impact these mismatches have in PCR.^15^

Characterizing the stability of the primer-template complex anchor region via the gold standard nearest-neighbor approach did not outperform positional classification. This result suggests that the position and nucleotides involved in a particular mismatch are more informative than the stability of the primer-template complex. Such an insight aligns with our current understanding of polymerase fidelity and the concept of active site tightness, which highlights the nucleotides involved in a mismatch govern the impact on the polymerase due to differences in steric hindrances.^33^ Further research is required to quantify the steric hindrance induced by different mismatch combinations and, subsequently, if this quantifiable parameter can be used to predict the impact of a given mismatch on RPA reaction kinetics.

Our investigation highlights the importance of considering variation in primer binding sites for RPA diagnostic applications, as a single mismatch has the potential to reduce the probability of reaction success to 0.589, compromising both sensitivity and specificity. Addressing the impact of mismatch on reaction success is a potential issue for robust SNP profiling. The introduction of specific mismatches needs to completely inhibit the reaction, enabling binary classification to indicate the presence or absence of a particular genotype. Within the scope of our investigation, the deliberate introduction of certain T1n mismatch combinations, such as ??CA-??CC and ??TC-??CC, completely inhibited the RPA reaction, whereas the corresponding single penultimate mismatches, ??C?-??C? and ??T?-??C?, only mildly retarded the RPA reaction kinetics. Alternatively, the RPA reaction could be designed to guarantee reaction success in the presence of mismatches, while maintaining heterogeneity in reaction kinetics, which could be used for SNP classification. The strong correlation between reaction kinetic metrics, such as TP and TMG, could be used to enhance the feasibility and reliability of a metric clustering approach to determine SNP presence. To achieve the desired changes in mismatch impact on reaction success, a variety of strand-displacing polymerases should be screened to identify those more sensitive and tolerant to mismatch combinations.

In summary, we have systematically investigated the impact of terminal mismatches on the RPA reaction across several clinically relevant biomarkers. Through implementing a range of statistical models, we have determined the impact of 315 mismatch combination on the RPA reaction, highlighting to RPA users the pitfalls of bad primer design and proving a foundation on which to build for RPA-based SNP genotyping. We hope that our description on RPA mismatch tolerance will form the foundation of improved RPA primer design using computational programs, such as PrimedRPA, aiding in the design of robust assays, especially in targets with high genetic diversity. The implementation of RPA-based SNP genotyping and diagnostics for infections and diseases, especially in high burden populations, has the potential to inform clinical and surveillance decision making, leading to personalized treatment of patients with improved outcomes and healthier populations.
